# Performance evaluation of automated urine microscopy as a rapid, non-invasive approach for the diagnosis of non-gonococcal urethritis

**DOI:** 10.1136/sextrans-2014-051761

**Published:** 2015-01-22

**Authors:** Marcus J Pond, Achyuta V Nori, Sheel Patel, Ken Laing, Margarita Ajayi, Andrew J Copas, Philip D Butcher, Phillip Hay, Syed Tariq Sadiq

**Affiliations:** 1 Institute for Infection and Immunity, St George's University of London, London, UK; 2 Department of Genitourinary & HIV Medicine, St George's Healthcare NHS Trust, London, UK; 3 Research Department of Infection and Population Health, University College London, Mortimer Market Centre, London, UK

**Keywords:** URETHRITIS, DIAGNOSIS, M GENITALIUM, CHLAMYDIA TRACHOMATIS, MOLECULAR TECHNIQUES

## Abstract

**Objectives:**

Gram-stained urethral smear (GSUS), the standard point-of-care test for non-gonococcal urethritis (NGU) is operator dependent and poorly specific. The performance of rapid automated urine flow cytometry (AUFC) of first void urine (FVU) white cell counts (UWCC) for predicting *Mycoplasma genitalium* and *Chlamydia trachomatis* urethral infections was assessed and its application to asymptomatic infection was evaluated.

**Methods:**

Receiver operating characteristic curve analysis, determining FVU-UWCC threshold for predicting *M. genitalium* or *C. trachomatis* infection was performed on 208 ‘training’ samples from symptomatic patients and subsequently validated using 228 additional FVUs obtained from prospective unselected patients.

**Results:**

An optimal diagnostic threshold of >29 UWC/µL gave sensitivities and specificities for either infection of 81.5% (95% CI 65.1% to 91.6%) and 85.8% (79.5% to 90.4%), respectively, compared with 86.8% (71.1% to 95%) and 64.7% (56.9% to 71.7%), respectively, for GSUS, using the training set samples. FVU-UWCC demonstrated sensitivities and specificities of 69.2% (95% CI 48.1% to 84.9%) and 92% (87.2% to 95.2%), respectively, when using validation samples. In asymptomatic patients where GSUS was not used, AUFC would have enabled more infections to be detected compared with clinical considerations only (71.4% vs 28.6%; p=0.03). The correlation between UWCC and bacterial load was stronger for *M. genitalium* compared with *C. trachomatis* (τ=0.426, p≤0.001 vs τ=0.295, p=0.022, respectively).

**Conclusions:**

AUFC offers improved specificity over microscopy for predicting *C. trachomatis* or *M. genitalium* infection. Universal AUFC may enable non-invasive diagnosis of asymptomatic NGU at the PoC. The degree of urethral inflammation exhibits a stronger association with pathogen load for *M. genitalium* compared with *C. trachomatis*.

## Introduction

Non-gonococcal urethritis (NGU) is diagnosed at the point of care (PoC) by demonstrating ≥5 polymorphonuclear cells per high power field (PMN/HPF) on a Gram-stained urethral smear (GSUS) or ≥10 PMN/HPF from first void urine (FVU) sample sediments.^1^ These thresholds were derived from studies predicting urethral infection prior to implementation of nucleic acid amplification testing (NAAT).^2^ ^3^ Taking urethral swabs for GSUS is uncomfortable for patients.^4^ Furthermore, microscopy requires skilled personnel and possesses significant inter-user and intra-user variability,^5^ ^6^ variable sensitivity and low specificity for predicting infection.^7^ Reduced use of microscopy for asymptomatic patients and the expansion of ‘test and treat’ community-based sexually transmitted infection screening programmes for *Chlamydia trachomatis* infection only reduce the potential for empirically treating other infective causes of NGU, such as *Mycoplasma genitalium*.

With a few accurate specific PoC tests for common microbiological causes of NGU available, a rapid, reproducible, cost-effective and accurate alternative to GSUS is required. Automated urine flow cytometry (AUFC) of mid-stream urine is widely adopted by microbiology laboratories for diagnosing urinary tract infections.^8–10^ This method may also be suitable for diagnosing urethritis if deployable near sexual health clinics, but this has not been evaluated. We compared the test performance of GSUS with AUFC of FVU specimens for predicting male urethral infection with either *M. genitalium* or *C. trachomatis* in cases of symptomatic and asymptomatic NGU.

## Patients and methods

### Training samples

Samples were collected as part of a previous study determining the prevalence of aetiological agents of urethritis.^11^ Men with symptoms suggestive of urethritis, prospectively presenting to the genitourinary medicine (GUM) clinic at St George's Healthcare National Health Service Trust between 28 September 2011 and 15 December 2011, undergoing routine GSUS were eligible for inclusion. Patients were designated as having either ‘urethritis’ or ‘no urethritis’, as described previously,^11^ but excluded if GSUS, NAAT or culture indicated the presence of *Neisseria gonorrhoeae*.

### Validation samples

All men attending GUM between 31 July 2013 and 15 September 2013. Following sample collection, only symptomatic patients underwent GSUS testing, a standard-stratified care pathway as GSUS of asymptomatic men reveals a few extra patients with urethritis.^12^ All patients underwent AUFC but were excluded if GSUS or subsequent NAAT or culture results indicated *N. gonorrhoeae* presence.

### Sample collection and processing

All subjects provided a FVU sample (10–50 mL), not having voided urine for at least 2 h, 2 mL of which was sent for routine *C. trachomatis* and *N. gonorrhoeae* NAAT with BD Viper Qx System (Becton Dickinson, Oxford, UK). A further 2 mL was sent immediately for AUFC. Following measurement of residual volume,^13^ nucleic acids were extracted from FVU as previously described.^11^ AUFC of FVU samples was performed using UF-100i urine flow cytometer (Sysmex, Milton Keynes, UK). Urinary white cell counts (UWCC) were enumerated per µL of urine.

### PCR

Endpoint real-time PCR was performed as described previously^2^ using probe sets enabling detection of *C. trachomatis*^14^ *and M. genitalium*.^15^ Droplet digital PCR (ddPCR) was used to determine pathogen genome copies per mL of original volume of FVU collected. Briefly, reaction mixtures of a final volume of 20 μL were generated in triplicate, each comprising 10 μL of ddPCR Master Mix (Bio-Rad), relevant forward and reverse primers and probe and 8 µL of extracted DNA. Droplets were generated using each 20 μL reaction and 60 μL of droplet generation oil using a droplet generator cartridge and droplet generator (Bio-Rad). Following dispersion, reactions were transferred into 96-well plates (Eppendorf, Stevenage, UK), heat sealed with foil and amplified using the following conditions: 10 min, 95°C; 40 cycles of 15 s at 94°C and 60 s at 60°C and 10 min final inactivation, 98°C. Fluorescence-positive droplets for each sample were enumerated using QuantaSoft software package and QX100 instrument (Bio-Rad).

### Statistical analysis

Previous work^7^ suggested that GSUS has 63% specificity for diagnosing *C. trachomatis*. The training set data for this study were drawn from a previously reported prevalence-of-infection study,^11^ with a sample size of approximately 100 samples from each of symptomatic non-urethritis and urethritis patients. This sample set was deemed suitable for the training set as it allowed an increase in specificity of UWCC over GSUS of 15% to be detected with >90% power, (α=0.05), but was too small to detect changes in sensitivity. The goal of our study was to investigate specificity improvements. In order to achieve a diagnostic specificity and 95% confidence range comparable to those observed in the training sample analysis, 220 prospective samples in total were collected for the validation set. Receiver operating characteristic (ROC) curves of AUFC performance for the prediction of either *C. trachomatis* or *M. genitalium* infection were derived from training set data. Non-parametric cumulative distribution functions for the ROC curves were drawn empirically and area under the curve was determined. Optimal thresholds of test positivity were defined using Youden's index of J.

For the training set, AUFC performance for predicting infection was compared with GSUS. For the validation set, in two planned additional analyses we first compared UWCC performance alone against combined use of UWCC with GSUS in order to explore the impact of retaining GSUS for *N. gonorrhoeae* detection in symptomatic patients; second, we focused on asymptomatic patients where GSUS is not used and where treatment decisions are based on clinical or epidemiological considerations alone and compared those infected with *C. trachomatis* or *M. genitalium* who received treatment with those who would have received treatment if UWCC were used to aid diagnosis at the PoC. The validation set was selected to show equivalent specificity and also derives information regarding the performance of the assay in routine use.

In these three analyses, comparisons were made using McNemar’s test for paired binary data. χ^2^ tests were used to detect differences in symptoms and pathogen prevalence between urethritis and non-urethritis groups within the training set. Correlations between discrete variables were assessed using Kendall tau rank correlation coefficient. UWCC and bacterial load distributions were compared using Mann-Whitney U test. Statistical analyses were performed using SPSS V.21.

This study was approved by Wandsworth Research Ethics Committee, London (project number: Q080371).

## Results

### Training set

FVU specimens were collected from 208 symptomatic patients (mean age 31 years), 104 with urethritis and 104 with non-urethritis defined clinically as above. As expected, symptoms of urethral discharge (69% vs 50%; p≤<0.001) and dysuria (45% vs 27%; p=0.009) were higher in the urethritis group compared with non-urethritis group, respectively, but there was no difference in proportions reporting discomfort (21% vs 27%, respectively; p=0.330).

Overall prevalence of urethral pathogens was 10.5% (n=22) for *M. genitalium* and 7.6% (n=16) for *C. trachomatis.* Both infections were more prevalent in those diagnosed with urethritis: *M. genitalium* was detected in 16.3% (n=17) of urethritis patients and 4.8% (n=5) of non-urethritis patients (p=0.007). *C. trachomatis* was detected in 14.4% (n=15) of urethritis cases and 1% (n=1) of non-urethritis cases (p≤0.001). No patients were dually infected with *M. genitalium* and *C. trachomatis*.

### First void UWCC and GSUS associated with urethral infection within the training set

**Figure 1:**
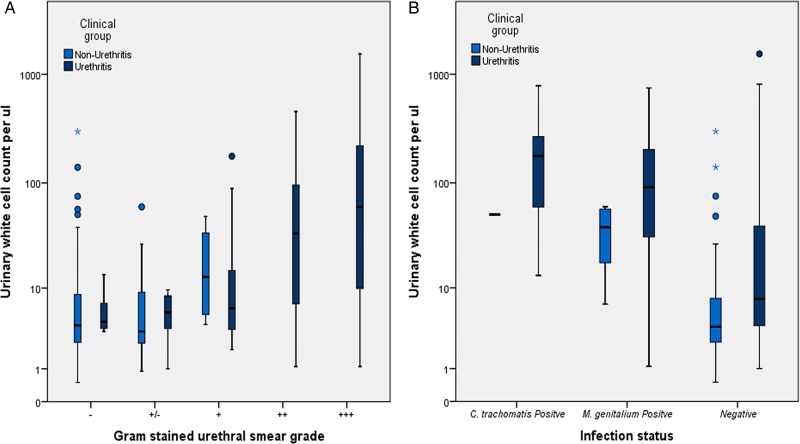
Box plots comparing the distributions of first void urinary white cell counts (UWCC) per μL in patients with and without urethritis stratified by urethral smear grade (A) and *C. trachomatis* or *M. genitalium* infection status (B). Training set patients only. The median urinary white cell count increases as the urethral smear grade increases (A, p≤0.001). UWCC are increased in infected patients with respect to non-infected patients (B, p≤0.001), regardless of clinical grouping. Negative Gram-stained urethral smear (GSUS): ‘−’ (0 PMN/HPF), ‘−/+’ (1–5 PMN/HPF). Positive GSUS: ‘+’ (5–10 PMN/HPF), ‘++’ (10–20 PMN/HPF) and ‘+++’ (>20 PMN/HPF). PMN/HPF, polymorphonuclear cells per high power field.

Grading of GSUSs demonstrated a difference in distribution between urethritis groups compared with non-urethritis groups (p≤0.001) (see figure 1). As expected, 68% (n=71) of patients in the urethritis group demonstrated more than 10 PMN/HPF (median >20 PMN/HPF). Within the non-urethritis group, 96.1% (n=100) of patients possessed less than 10 PMN/HPF (median <5 PMN/HPF). Four patients in the non-urethritis group had a microscopy grade of 5–10 PMN/HPF, but were included in the non-urethritis group on clinical grounds.

UWCC measured by AUFC were increased in the urethritis group (median 13.7 UWCC/µL) when compared with the non-urethritis group (median 4 UWCC/µL; p≤0.001). A correlation between increasing UWCC and GSUS grade was observed (see figure 1, τ=0.374, p<0.001). No correlation between the volume of FVU received and UWCC/µL was observed (τ=−0.059, p=0.201).

Patients infected with either *C. trachomatis* or *M. genitalium* had increased GSUS grades and median UWCC compared with non-infected patients (10–20 PMN/HPF vs 1–5 PMN/HPF, respectively, p<0.001; and 73 UWCC/µL vs 5 UWCC/µL, respectively, p≤0.001, see figure 1B).

### Assignment of threshold to predict urethral infection from UWCC

In order to investigate the diagnostic utility of AUFC-UWCC, an ROC curve was plotted (see online supplementary figure S3). Youden's index analysis gave an indicative threshold for predicting either *C. trachomatis* or *M. genitalium* infection of ≥29 UWCC/µL (Youden's index=0.675). At this threshold, UWCC had a similar sensitivity to GSUS, 81.5% (95% CI 65.1% to 91.6%) versus 86.8% (95% CI 71.1% to 95%), respectively, and significantly improved specificity (p<0.001) and positive predictive value (85.8% (95% CI 79.5% to 90.5%) vs 64.7% (95%CI 56.9% to 71.7%) respectively; see table 1).

**Table 1:** Comparison of FVU-UWCC with GSUS test performance for the prediction of either *Chlamydia trachomatis* or *Mycoplasma genitalium* infection using training set samples

Test organism	Sensitivity		p Value*	Specificity		p Value*	Positive predictive value		Negative predictive value	
	UWCC†	GSUS‡		UWCC†	GSUS‡		UWCC†	GSUS‡	UWCC†	GSUS‡
*C. trachomatis*	93.7% (67.7% to 99.6%)	93.7% (67.7% to 99.6%)	1	N/A	N/A		28.3% (17.1% to 42.5%)	16.1% (9.6% to 25.5%)	99.3% (95.8% to 99.9%)	99.1% (94.5% to 99.9%)
*M. genitalium*	72.7% (49.5% to 88.3%)	81.8% (58.9% to 94%)	0.72	N/A	N/A		29% (18% to 43%)	19.3% (12.1% to 29.1%)	96% (91.2% to 98.3%)	96.5% (90.8% to 98.8%)
*C. trachomatis* or *M. genitalium*	81.5% (65.1% to 91.6%)	86.8% (71.1% to 95%)	0.75	85.8% (79.5% to 90.4%)	64.7% (56.9 to 71.7%)	<0.001	56.3% (42.3% to 69.4%)	35.4% (26% to 46.1%)	95.4% (90.4% to 97.9%)	95.6% (89.6% to 98.3%)

*p Values represent the results of statistical comparisons of either sensitivity or specificity between the two test methodologies. These were generated using McNemar’s test for paired binary data with the results of *C. trachomatis* NAAT testing upon BD Viper System or real-time PCR for *M. genitalium* as the reference standards.

†UWCC were defined as positive if the patient’s urine contained >29 White blood cell per µL.

‡GSUS testing containing >5 PMN per high power fields (HPFs) over 5 HPFs was examined.

FVU, first void urine; GSUS, Gram-stained urethral smear; NAAT, nucleic acid amplification testing; UWCC, urinary white cell counts.

### Validation set

Of 228 patients providing FVU specimens (mean age 32 years), 22% (n=52) were symptomatic and therefore had GSUS, and 9% (n=20) demonstrated >10 PMN/HPF. Overall *C. trachomatis* and *M. genitalium* prevalence was 6% (n=14) and 5% (n=12), respectively. Among symptomatic patients, *M. genitalium* and *C. trachomatis* prevalence was 8% (n=4) and 17% (n=9), respectively, and among asymptomatic patients was 3% (n=5) and 5% (n=8), respectively. No correlation was observed between FVU volume and UWCC (τ=−0.070, p=0.120).

### First void UWCC test performance for prediction of *C. trachomatis* or *M. genitalium* infection

The ≥29 UWCC/µL threshold derived from the training set was applied to the independent validation set. Sensitivities, specificities and predictive values are shown in table 2. As expected, there was a reduction in sensitivity compared with the training set, although specificity remained high.

**Table 2:** Diagnostic performances of UWCC, parallel UWCC/GSUS and stratified diagnosis for the prediction of *Chlamydia trachomatis* or *Mycoplasma genitalium* infection using validation samples

Diagnostic performance comparison of UWCC versus UWCC and GSUS performed in parallel using validation samples (n=228)										
	UWCC* sensitivity	Parallel UWCC* and GSUS† sensitivity	p Value‡	UWCC* specificity	Parallel UWCC* and GSUS† specificity	p Value‡	UWCC* PPV§	Parallel UWCC and GSUS PPV§	UWCC* NPV¶	Parallel UWCC and GSUS NPV¶
*C. trachomatis*	71.4% (42 to 90.4)	78.6% (48.8 to 94.3)	0.5	N/A	N/A		29.4% (15.7 to 47.6)	26.8% (14.8 to 43.2)	97.9% (94.4 to 99.3)	98.3% (95 to 99.5)
*M. genitalium*	66.6% (35.4 to 88.7)	66.60% (35.4 to 88.7)	0.25	N/A	N/A		23.5% (11.3 to 41.5)	19.5% (93 to 35.3)	97.9% (94.4 to 99.3)	97.9% (94.3 to 99.3)
*C. trachomatis* or *M. genitalium*	69.2% (48.1 to 84.9)	73% (51.9 to 87.6)	0.031	92% (87.2 to 95.2)	89.1% (83.7 to 92.8)	0.03	53% (35.3 to 69.8)	46.3% (30.9 to 60.3)	95.8% (91.7 to 98)	96.2% (92.1 to 98.3)

Diagnostic performance comparison of UWCC versus stratified diagnosis using validation samples of asymptomatic patients only (n=176)										
	UWCC* sensitivity	Stratified diagnosis** sensitivity	p Value‡	UWCC* specificity	Stratified diagnosis** specificity	p Value‡	UWCC* PPV§	Stratified diagnosis PPV§	UWCC* NPV¶	Stratified approach NPV¶
*C. trachomatis*	100% (40.3% to 100%)	60% (17% to 92.7%)	0.5	N/A	N/A		19.2% (7.3% to 39.9%)	30% (8% to 64.6%)	100% (96.8% to 100%)	98.8% (95.3% to 99.8%)
*M. genitalium*	50% (17.4% to 82.6%)	12.5% (0.6% to 53.3%)	0.25	N/A	N/A		15.4% (5% to 35.7%)	10% (0.5% to 45.9%)	97.3% (92.9% to 99.1%)	95.8% (91.1% to 98.1%)
*C. trachomatis* or *M. genitalium*	71.4% (42% to 90.4%)	28.6% (9.6% to 58%)	0.031	90.1% (84.2% to 94%)	96.3% (91.8% to 98.5%)	<0.01	38.5% (20.9% to 59.3%)	40% (13.7% to 72.6%)	97.3% (92.9% to 99.1%)	93.8% (88.9% to 96.9%)

*UWCC were defined as positive if the patient’s urine contained >29 White blood cell per µL.

†GSUS testing containing >5 PMN per high power fields (HPFs) over 5 HPFs was examined.

‡p Values represent the results of statistical comparisons of either sensitivity or specificity between the two test methodologies. These were generated using McNemar’s test for paired binary data with the results of *C. trachomatis* NAAT testing upon BD Viper System or real-time PCR for *M. genitalium* as the reference standards.

§Positive predictive value.

¶Negative predictive value.

**Positive stratified diagnosis is defined as patients treated for urethritis due to suggestive clinical history.

GSUS, Gram-stained urethral smear; NAAT, nucleic acid amplification testing; UWCC, urinary white cell counts.

Additional analysis demonstrated that combining UWCC with GSUS for symptomatic patients resulted in slightly decreased test specificity (89.1% vs 92%, p=0.03) when compared with UWCC alone (see table 2).

In asymptomatic patients (n=176), combining UWCC testing with epidemiological suspicion of infection and subsequent treatment improved sensitivity (71.4% vs 28.6%, p=0.031), but reduced specificity (90.1% vs 96.3%, p=0.031) when compared with combining epidemiological suspicion and treatment alone (see table 2).

### Associations between bacterial load and UWCC

There was no difference in FVU bacterial loads between *M. genitalium*-infected patients (n=34) and *C. trachomatis*-infected patients (n=30), derived from both training and validation sets (median 256.9 copies/mL cf 194.7 copies/mL, respectively; p=0.389). The median FVU bacterial load, whether combined or for each organism, was higher in those with positive UWCC tests (≥29 UWCC/mL), compared with those with negative UWCC tests (470 copies/mL *cf* 36.8 copies/mL, respectively, for *M. genitalium* and *C. trachomatis* combined, p=0.001; 538.7 copies/mL *cf* 91.1 copies/mL, respectively, for *M. genitalium* only, p=0.02; 306.9 copies/mL *cf* 15.4 copies/mL, respectively, for *C. trachomatis* only, p=0.005). UWCC were higher in *C. trachomatis*-infected patients compared with *M. genitalium*-infected patients (median, 83.7 UWCC/µL *cf* 49 UWCC/µL, respectively, p=0.024). UWCC were positively correlated with *M. genitalium* bacterial load (see online supplementary figure S2A, τ=0.426, p≤0.001) and more weakly correlated with *C. trachomatis* bacterial load (see online supplementary figure S2B, τ=0. 295, p=0.022). Bacterial loads were not statistically different between GSUS-positive and GSUS-negative patients, whether asymptomatic or symptomatic (median 305.5 copies/mL *cf* 139.9 copies/mL, respectively; p=0.58).

## Discussion

We observed that AUFC of total white cell count from first void male urine samples, using a small bench-top AUFC system, suitable for implementation within or near GUM clinic settings, improved performance for predicting either *C. trachomatis* or *M. genitalium* urethritis in comparison with GSUS, counting neutrophils per HPF. The automated urine test has the potential to identify patients with asymptomatic urethritis in a non-invasive manner.

A UWCC threshold of >29/µL applied to symptomatic patients demonstrated improved specificity (85.8% vs 64.7%) and positive predictive value (56.3% vs 35.4%) when compared with GSUS for predicting either infection. Subsequent testing on an independent cohort of men, the majority of whom were asymptomatic, validated these findings albeit with a possible slight but expected reduction in specificity when UWCC were combined with GSUS. Our data suggest AUFC represents an improved PoC test for NGU when compared with microscopy for predicting urethritis caused by C. *trachomatis* or *M. genitalium*. However, first-line treatments for the two pathogens are different. In the absence of pathogen-specific PoC tests, positive AUFC might best be used as per NGU management guidelines or as prescreening for using pathogen-specific PoC tests when the latter become available.

Associations between PMN leucocyte counts present within FVU sediment and urethral exudate specimens were first observed by Bowie.^2^ More recently, microscopic enumeration of antibody-stained white cells within FVU s was used to study the association between leucocyte counts and urethral pathogens,^16^ a technique unsuitable for high throughput use. GSUS has therefore remained predominantly in use for diagnosing urethritis on account of its technical simplicity.

Compared with GSUS, AUFC is simple to perform, rapid (within a few minutes), offers increased throughput and obviates the need for invasive urethral swabbing. These features will improve patient acceptability and potentially allow for triaging patients into those with and without urethritis on entering the clinic. Incorporation of further improvements in portability of flow cytometric devices^17^ may allow for use in outreach settings, enabling many more patients to be screened rapidly for urethritis without the need to attend tertiary healthcare for diagnosis.

Preliminary experiments carried out alongside this study (data not shown) suggest AUFC is capable of detecting inflammation caused by gonococcal infection. A potential pitfall of replacing GSUS with AUFC is the loss of PoC diagnosis of *N. gonorrhoeae* infection. Observation of intracellular Gram-negative diplococci upon GSUS possesses sensitivities and specificities approaching 100% for the diagnosis of gonorrhoea.^18^ Our results suggest continuing to deploy GSUS in symptomatic patients alongside UWCC testing for all patients would retain PoC *N. gonorrhoeae* detection while maximising the sensitivity of screening for *C. trachomatis* and *M. genitalium*. Future application of AUFC in parallel with accurate PoC NAAT testing for gonorrhoea^19^ should overcome the requirement to retain GSUS.

The sensitivity of GSUS in predicting chlamydial infection in our study was 93.7% in comparison with 23–63% in previous studies using NAATs, a difference possibly attributable to increased sensitivity of the *C. trachomatis* test platform we used (BD Viper NAAT)^20^ when compared with these other studies.^7^ ^21^ Our study was not powered to demonstrate a difference in *sensitivity* between GSUS and AUFC, although we believe the benefits in specificity and the non-invasive nature of AUFC represent real clinical advantages. The specificity of AUFC was likely compromised because we evaluated the accuracy of predicting *C. trachomatis* or *M. genitalium* infections, together which usually account for half of NGU causes. All study samples were screened for *Trichomonas vaginalis* but a very low prevalence was observed (data not shown), precluding its inclusion in this study. Wider population-based studies characterised for larger sets of known^22^ and candidate^23^ causes of NGU may allow further assessment of AUFC test performance.

Asymptomatic patients did not form part of training set samples and therefore the identified FVU-UWCC threshold may not be optimal for identification of urethral infection in these patients. Inclusion of asymptomatic patients within training samples would have required them to receive unnecessary and potentially uncomfortable GSUS testing, which is not recommended by current guidelines. In this study we applied Youden's index of J, a methodology that applies equal weighting to both sensitivity and specificity when determining the threshold of diagnostic test positivity. In future, separately developed thresholds for asymptomatic patients may be necessary for maximum test performance, with potentially greater weight given to specificity to prevent inappropriate overtreatment of patients.

GSUS and AUFC results did not demonstrate absolute congruence. Four *M. genitalium-*infected patients and three *C. trachomatis-*infected patients were positive by GSUS but demonstrated <29 UWCC/µL by AUFC. Bacterial loads for both organisms in these patients were significantly lower than the remaining infected patients, suggesting AUFC may lack sensitivity for predicting infections with a low bacterial burden. Five patients infected with *M. genitalium* and two with *C. trachomatis* were positive using AUFC but negative by GSUS, perhaps attributable to technical and observer variations in GSUS.^5^ These patients did not demonstrate reduced bacterial loads. Within validation samples, 13 *M. genitalium*-positive patients or *C. trachomatis*-positive patients did not undergo microscopy as they were asymptomatic and not required to do so.^12^ Three of these patients were asymptomatic contacts of CT and of these two were UWCC positive.

Associations between bacterial load and UWCC gave insight into pathogenesis and potential interactions with other infections. First, we demonstrated *C. trachomatis-*infected patients possessed significantly higher UWCC when compared with *M. genitalium*-positive patients. In order to further investigate this finding, we assessed the relationship between UWCC and pathogen load. Bacterial load range of *M. genitalium* was comparable with other studies^24^ ^25^ and there was no difference in pathogen loads between *M. genitalium* and *C. trachomatis*, although this analysis was a secondary outcome of the study and may not have been sufficiently powered to demonstrate this. However, UWCC were higher in *C. trachomatis* infection compared with *M. genitalium*, suggesting different interactions or effects of inflammation on bacterial replication in the male genital tract between the two organisms.^26^ ^27^ Second, *C. trachomatis* bacterial loads have been shown to be present at 100-fold greater concentrations than *M. genitalium* on vaginal swabs, and higher in vaginal swabs compared with male FVU^28^ ^29^ samples. Our findings of similar loads of *C. trachomatis* and *M. genitalium* within male FVU samples perhaps suggest an altered pathogenesis between male and female infections. Finally, the observation of a strong proportionate relationship between bacterial burden and inflammatory response observed in this study for *M. genitalium* supports recent observations that levels of genital HIV secretion are increased when *M. genitalium* burden is high.^30^

In conclusion, in the absence of PoC tests capable of detecting common causes of NGU, and decreased use of GSUS, implementation of automated UWCC may be an attractive, accurate, PoC screening test for urethritis and urethral infection in symptomatic and asymptomatic men, enabling immediate syndromic management for a greater number of patients infected with either *C. trachomatis* or *M. genitalium*.

**Key messages**

- Automated flow cytometric analysis of first void urine specimens for the presence of elevated urine white cell counts (UWCCs) is a practical method for screening patients for the detection of urethral infection.
- This technique is non-invasive, user-independent, rapid and has enhanced diagnostic specificity when compared with gram-stained urethral smear.
- Application of this test may allow for and improve the diagnosis of urethritis in patients who do not report symptoms.
- UWCC are related to pathogen load and may be useful in field and community studies of patients with STIs.

## Supplementary Material

Web supplement

## References

[1] Martin DH. Urethritis in males. In: Holmes KK, Sparling PF, Stamm WE, *et al*, eds. Sexually Transmitted Diseases. 4th edn. New York: McGraw-Hill, 2008:1107–27.

[2] Bowie WR Comparison of Gram stain and first-voided urine sediment in the diagnosis of urethritis. Sex Transm Dis 1978;5:39–42. 10328029 10.1097/00007435-197804000-00001

[3] Swartz SL, Kraus SJ, Herrmann KL, et al Diagnosis and etiology of nongonococcal urethritis. J Infect Dis 1978;138:445–54. 213495 10.1093/infdis/138.4.445

[4] Apoola A, Herrero-Diaz M, FitzHugh E, et al A randomised controlled trial to assess pain with urethral swabs. Sex Transm Infect 2011;87:110–13. 21131307 10.1136/sti.2010.042861

[5] Smith R, Copas AJ, Prince M, et al Poor sensitivity and consistency of microscopy in the diagnosis of low grade non-gonococcal urethritis. Sex Transm Infect 2003;79:487–90. 14663127 10.1136/sti.79.6.487 PMC1744776

[6] Willcox JR, Adler MW, Belsey EM Observer variation in the interpretation of Gram-stained urethral smears: implications for the diagnosis of non-specific urethritis. Br J Vener Dis 1981;57:134–36. 6163500 10.1136/sti.57.2.134 PMC1045889

[7] Haddow LJ, Bunn A, Copas AJ, et al Polymorph count for predicting non-gonococcal urethral infection: a model using *Chlamydia trachomatis* diagnosed by ligase chain reaction. Sex Transm Infect 2004;80:198–200. 15170002 10.1136/sti.2003.006924 PMC1744835

[8] Broeren MA, Bahceci S, Vader HL, et al Screening for urinary tract infection with the Sysmex UF-1000i urine flow cytometer. J Clin Microbiol 2011;49:1025–29. 21248088 10.1128/JCM.01669-10 PMC3067737

[9] Jolkkonen S, Paattiniemi EL, Karpanoja P, et al Screening of urine samples by flow cytometry reduces the need for culture. J Clin Microbiol 2010;48:3117–21. 20592157 10.1128/JCM.00617-10 PMC2937741

[10] Pieretti B, Brunati P, Pini B, et al Diagnosis of bacteriuria and leukocyturia by automated flow cytometry compared with urine culture. J Clin Microbiol 2010;48:3990–6. 20739491 10.1128/JCM.00975-10 PMC3020858

[11] Pond MJ, Nori AV, Witney AA, et al High prevalence of antibiotic-resistant *Mycoplasma genitalium* in nongonococcal urethritis: the need for routine testing and the inadequacy of current treatment options. Clin Infect Dis 2014;58:631–7. 24280088 10.1093/cid/cit752 PMC3922211

[12] British Association for Sexual Health and HIV. Sexually Transmitted Infections: UK national screening and testing guidelines. 31-8-2006. 1-8-2006.

[13] Naccarato WF, Treuting JJ, Cannon DC Gravimetric determination of urine volumes. Am J Med Technol 1981;47:111–12. 7223757

[14] Schaeffer A, Henrich B Rapid detection of *Chlamydia trachomatis* and typing of the Lymphogranuloma venereum associated L-Serovars by TaqMan PCR. BMC Infect Dis 2008;8:56. 18447917 10.1186/1471-2334-8-56 PMC2387162

[15] Jensen JS, Bjornelius E, Dohn B, et al Use of TaqMan 5′ nuclease real-time PCR for quantitative detection of *Mycoplasma genitalium* DNA in males with and without urethritis who were attendees at a sexually transmitted disease clinic. J Clin Microbiol 2004;42:683–92. 14766837 10.1128/JCM.42.2.683-692.2004 PMC344445

[16] Wiggins RC, Holmes CH, Andersson M, et al Quantifying leukocytes in first catch urine provides new insights into our understanding of symptomatic and asymptomatic urethritis. Int J STD AIDS 2006;17:289–95. 16643676 10.1258/095646206776790268

[17] Moon S, Gurkan UA, Blander J, et al Enumeration of CD4+ T-cells using a portable microchip count platform in Tanzanian HIV-infected patients. PloS One 2011;6:e21409. 21754988 10.1371/journal.pone.0021409 PMC3130745

[18] Taylor SN, DiCarlo RP, Martin DH Comparison of methylene blue/gentian violet stain to Gram's stain for the rapid diagnosis of gonococcal urethritis in men. Sex Transm Dis 2011;38:995–96. 21992973 10.1097/OLQ.0b013e318225f7c2

[19] Gaydos CA, Van Der PB, Jett-Goheen M, et al Performance of the Cepheid CT/NG Xpert rapid PCR test for detection of *Chlamydia trachomatis* and *Neisseria gonorrhoeae*. J Clin Microbiol 2013;51:1666–72. 23467600 10.1128/JCM.03461-12 PMC3716060

[20] Taylor SN, Van Der PB, Lillis R, et al Clinical evaluation of the BD ProbeTec *Chlamydia trachomatis* Qx amplified DNA assay on the BD Viper system with XTR technology. Sex Transm Dis 2011;38:603–9. 21301389 10.1097/OLQ.0b013e31820a94d2 PMC3763498

[21] Orellana MA, Gomez-Lus ML, Lora D Sensitivity of Gram stain in the diagnosis of urethritis in men. Sex Transm Infect 2012;88:284–87. 22308534 10.1136/sextrans-2011-050150

[22] Bradshaw CS, Tabrizi SN, Read TR, et al Etiologies of nongonococcal urethritis: bacteria, viruses, and the association with orogenital exposure. J Infect Dis 2006;193:336–45. 16388480 10.1086/499434

[23] Manhart LE, Khosropour CM, Liu C, et al Bacterial vaginosis-associated bacteria in men: association of *Leptotrichia/Sneathia* spp. with nongonococcal urethritis. Sex Transm Dis 2013;40:944–9. 24220356 10.1097/OLQ.0000000000000054 PMC4188452

[24] Dupin N, Bijaoui G, Schwarzinger M, et al Detection and quantification of *Mycoplasma genitalium* in male patients with urethritis. Clin Infect Dis 2003;37:602–5. 12905147 10.1086/376990

[25] Muller EE, Venter JM, Magooa MP, et al Development of a rotor-gene real-time PCR assay for the detection and quantification of *Mycoplasma genitalium*. J Microbiol Methods 2012;88:311–15. 22230235 10.1016/j.mimet.2011.12.017

[26] Falk L, Fredlund H, Jensen JS Symptomatic urethritis is more prevalent in men infected with *Mycoplasma genitalium* than with *Chlamydia trachomatis*. Sex Transm Infect 2004;80:289–93. 15295128 10.1136/sti.2003.006817 PMC1744873

[27] Leung A, Eastick K, Haddon LE, et al *Mycoplasma genitalium* is associated with symptomatic urethritis. Int J STD AIDS 2006;17:285–88. 16643675 10.1258/095646206776790231

[28] Jalal H, Verlander NQ, Kumar N, et al Genital chlamydial infection: association between clinical features, organism genotype and load. J Med Microbiol 2011;60:881–88. 21415209 10.1099/jmm.0.028076-0

[29] Michel CE, Sonnex C, Carne CA, et al *Chlamydia trachomatis* load at matched anatomic sites: implications for screening strategies. J Clin Microbiol 2007;45:1395–02. 17376879 10.1128/JCM.00100-07 PMC1865904

[30] Manhart LE, Mostad SB, Baeten JM, et al High *Mycoplasma genitalium* organism burden is associated with shedding of HIV-1 DNA from the cervix. J Infect Dis 2008;197:733–36. 18266605 10.1086/526501 PMC4090222

