# Supplementary material for: Performance evaluation of automated urine microscopy as a rapid, non-invasive approach for the diagnosis of non-gonococcal urethritis
Source: Sex Transm Infect. 2015 Jan 22;91(3):165–70. doi: 10.1136/sextrans-2014-051761 (PMC4413809; doi:10.1136/sextrans-2014-051761)
Supplement: Web supplement [file sextrans-2014-051761-s1.pdf]

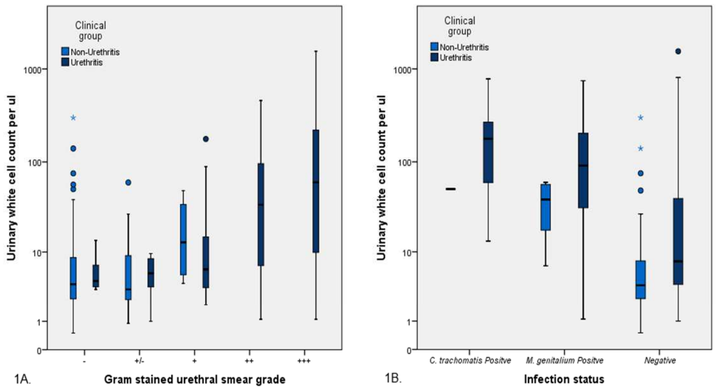

Figure 1. Boxplots comparing the distributions of first void urinary white cell counts per µL in patients with and without urethritis stratified by urethral smear grade (1A) and *C. trachomatis* or *M. genitalium* infection status (1B). Training set patients only. The median urinary white cell count increases as the urethral smear grade increases (1A, P=<0.001). UWCC counts are increased in infected patients with respect to non-infected (1B, p=<0.001), regardless of clinical grouping. Negative GSUS: '-'(0 PMN/HPF), '-/+' (1-5 PMN/HPF). Positive GSUS: '+' (5-10 PMN/HPF), '++' (10-20PMN/HPF) and '+++' (>20PMN/HPF).

1541x657mm (96 x 96 DPI)

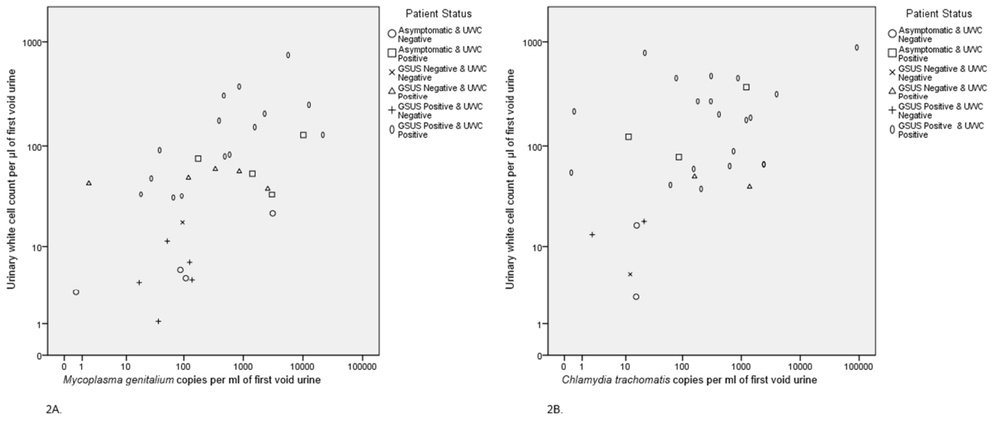

Supplementary Figure 2. First void UWCC count in relation to M. genitalium (2A) or C. trachomatis (2B) urinary load. Kendall tau b rank correlation observed and associations between UWCC count and M. genitalium urinary load (2A, $n=34$, $\tau=0.426$, $p=<0.001$) or C. trachomatis urinary load (2B, $n=30$, $\tau=0.333$, $p=0.10$)
331x158mm (96 x 96 DPI)

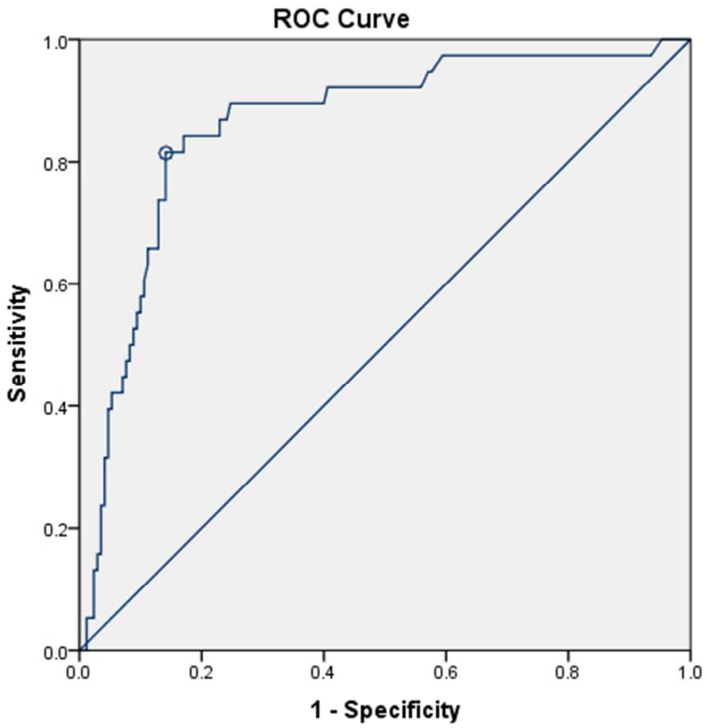

Supplemental Figure 3. Diagnostic receiver operator characteristic curve for the prediction of urethral infection from urinary white cell count. The y axis is the sensitivity and the x axis is 1-specificty. The green line is the reference line and the blue line is the test under analysis. The area under the curve is 0.855 (p=<0.001). Circle present upon ROC curve represents the threshold identified by Youden index analysis.
165x132mm (96 x 96 DPI)
